# Chlorogenic Acid Attenuates Bleomycin-Induced Pulmonary Fibrosis in a Murine Model by Modulating TGF-β1 Expression

**DOI:** 10.3390/arm94050060

**Published:** 2026-08-25

**Authors:** Juan Manuel Velázquez-Enríquez, Alma Aurora Ramírez-Hernández, Jovito César Santos-Álvarez, Edilburga Reyes-Jiménez, Antonio Arcos-Román, Jaime Arellanes-Robledo, Carlos Alberto Matias-Cervantes, María del Socorro Pina-Canseco, Verónica Rocío Vásquez-Garzón, Rafael Baltiérrez-Hoyos

**Affiliations:** 1 Laboratorio de Biomedicina Traslacional y Sistemas, Centro de Investigación en Nutrición y Alimentación, Universidad del Istmo, Carretera Transísmica Juchitán, La Ventosa km. 14, La Ventosa C.P. 70102, Oaxaca, Mexico; 2 Laboratorio de Fibrosis y Cáncer, Facultad de Medicina y Cirugía, Universidad Autónoma Benito Juárez de Oaxaca, Ex Hacienda de Aguilera S/N, Sur, San Felipe del Agua, Oaxaca de Juárez C.P. 68020, Oaxaca, Mexico; aramih_09@cecad-uabjo.mx (A.A.R.-H.); jovitocesarsa@cecad-uabjo.mx (J.C.S.-Á.); edilreyesjimnez@cecad-uabjo.mx (E.R.-J.); vrvasquezga@secihti.mx (V.R.V.-G.); 3 Laboratorio de Diagnóstico Molecular, Facultad de Medicina, Universidad Autónoma de Guerrero, Avenida Solidaridad S/N Hornos Insurgentes, Acapulco C.P. 39300, Guerrero, Mexico; 4 Laboratorio de Enfermedades Hepáticas, Instituto Nacional de Medicina Genómica—INMEGEN, Mexico City C.P. 14610, Mexico; jarellanes@inmegen.gob.mx; 5 Dirección Adjunta de Investigación Humanística y Científica, Consejo Nacional de Humanidades, Ciencias y Tecnologías—SECIHTI, Mexico City C.P. 03940, Mexico; 6 SECIHTI-Facultad de Medicina y Cirugía, Universidad Autónoma Benito Juárez de Oaxaca, Ex Hacienda de Aguilera S/N, Sur, San Felipe del Agua, Oaxaca C.P. 68020, Mexico; carloscervantes.ox@outlook.com; 7 Centro de Investigación Facultad de Medicina UNAM-UABJO, Facultad de Medicina y Cirugía, Universidad Autónoma “Benito Juárez” de Oaxaca, Oaxaca de Juárez C.P. 68120, Oaxaca, Mexico

**Keywords:** chlorogenic acid, pulmonary fibrosis, bleomycin, inflammation, TGF-β1, collagen, fibrosis

## Abstract

**Highlights:**

**What are the main findings?**

- Chlorogenic acid prevents alveolar architecture collapse and reduces total cell infiltration in bleomycin-challenged mice.
- Chlorogenic acid administration significantly suppressed collagen deposition, TGF-β1 expression, and α-SMA-positive myofibroblast activation.

**What are the implications of the main findings?**

- The effect of chlorogenic acid on TGF-β1 expression suggests a potential influence on its signaling pathway, highlighting its usefulness as a new natural therapeutic strategy to mitigate progressive pulmonary fibrosis.
- These findings support further exploration of dietary polyphenols to improve clinical outcomes and quality of life in patients with interstitial lung diseases.

**Abstract:**

Background: Idiopathic pulmonary fibrosis (IPF) is a progressive interstitial lung disease driven by aberrant extracellular matrix deposition, with the activity of transforming growth factor-beta 1 (TGF-β1) orchestrating fibrogenesis. Current therapies are limited by severe adverse effects, highlighting the unmet need for safer treatments. In this study, the therapeutic potential of chlorogenic acid (CGA) in a murine model of pulmonary fibrosis was evaluated. Methods: Pathology was induced on day 0 via subcutaneous osmotic minipumps delivering bleomycin (BLM) for one week, followed by pump removal on day 10. Therapeutic interventions with intragastric CGA (60 mg/kg) were administered daily from days 14 to 20. Lung tissue samples obtained on day 21 were analyzed via histological, immunohistochemical, and RT–PCR methods. Results: Histological analysis via H&E and Masson’s trichrome staining revealed that CGA treatment significantly attenuated alveolar thickening, restored the alveolar space, and reduced the total cell density and Ashcroft fibrosis score. Furthermore, CGA markedly decreased collagen deposition. Quantitative RT–PCR and immunohistochemical assays revealed that CGA effectively decreased the expression of Col1a1, TGF-β1, and the myofibroblast marker alpha-smooth muscle actin (α-SMA). Conclusions: CGA exerts important therapeutic effects by decreasing the expression of TGF-β1 and the activation of myofibroblasts, indicating that this natural polyphenol is a promising candidate for mitigating the progression of pulmonary fibrosis.

## 1. Introduction

Interstitial lung diseases (ILDs) represent a diverse group of disorders that primarily affect the pulmonary interstitium, and are characterized by chronic inflammation and progressive remodeling of lung tissue [1]. These conditions can severely impair respiratory function because of the aberrant extracellular matrix (ECM) deposition and the development of fibrotic tissue, which reduce the elasticity of the lung and alter its structural integrity, ultimately resulting in progressive respiratory impairment [1,2,3]. As the predominant and most destructive subtype of interstitial lung disease (ILD), idiopathic pulmonary fibrosis (IPF) poses a major clinical challenge [1]. IPF is a chronic and progressively debilitating disease, with an approximate incidence of 0.09 to 1.30 cases and a prevalence of 0.33 to 4.51 cases per 10,000 individuals worldwide [4]. It is more common in men and is usually diagnosed between the fifth and sixth decades of life [5,6]. Following diagnosis, the disease carries a discouraging outlook, with typical patient survival spanning only two to five years [7,8,9].

At the pathological level, IPF is characterized by a profound alteration in the balance between alveolar epithelium injury and tissue repair [2,10]. Recurrent epithelial damage, driven by environmental exposures (such as cigarette smoke or occupational pollutants), infections, autoimmune processes, and genetic susceptibility, promotes aberrant wound healing responses and contributes to the establishment of a persistent profibrotic microenvironment [11,12,13].

In this pathophysiological microenvironment, transforming growth factor-beta 1 (TGF-β1) acts as the primary molecular driver governing the fibrotic cascade [14,15,16]. Following tissue damage, the binding of TGF-β1 to its specific receptors triggers the activation of intracellular signaling cascades divided into the canonical pathway (mediated by Smad2/3) and alternative noncanonical pathways (including MAPK/ERK, p38, and PI3K/Akt) [15,16,17]. The convergence of these signals promotes the transcription of profibrotic genes that drive the differentiation of resident fibroblasts into contractile myofibroblasts [15,17,18]. These cells are primarily responsible for the uncontrolled collagen production, the formation of fibrous foci, and the irreversible loss of alveolar architecture [15,16,19,20]. Owing to its critical role in disease progression, modulation of TGF-β1 expression represents one of the most promising therapeutic strategies to slow the progression of IPF [16,21,22].

However, despite significant advances in understanding these molecular mechanisms, translating this knowledge into effective clinical options remains challenging [22,23,24,25]. Although approved clinical interventions effectively attenuate the rate of physiological decline, they remain insufficient to restore or reverse established parenchymal structural damage [23,26,27]. Furthermore, long-term management with these drugs is severely limited by a high incidence of gastrointestinal and systemic adverse effects, which can compromise patients’ quality of life and result in high rates of treatment discontinuation [25,27,28]. Since current advances in IPF have not yet achieved optimal quality of life or clinical outcomes, there is an unmet need for effective and tolerable therapies.

Renowned for its potent antioxidant and anti-inflammatory activities, the polyphenol chlorogenic acid (CGA) has demonstrated encouraging anti-fibrotic efficacy across diverse preclinical disease models [29,30]. As a case in point, the therapeutic intervention with CGA in mice subjected to ischemia/reperfusion (I/R) kidney injury effectively suppressed the inflammatory cascade by lowering the levels of key factors, exemplified by TLR-4, TNF-α, NF-κB, and MCP-1. Furthermore, it modulates the fibrogenic pathway by significantly reducing TGF-β1 expression and the formation of myofibroblasts [31]. Similarly, CGA treatment significantly reduced liver inflammation and fibrosis in a CCl4-induced model. This was achieved by modulating the activity of the TGF-β1/miR-21/Smad7 signaling pathway. Furthermore, it decreased the levels of profibrotic mediators such as CTGF, α-SMA, and TIMP-1 while increasing the expression of Smad7 and MMP-9, thereby promoting collagen degradation and inhibiting the profibrotic cascade [32]. In a murine model of bleomycin (BLM)-induced pulmonary fibrosis, treatment with CGA resulted in a significant reduction in pulmonary inflammation and fibrosis in a dose-dependent manner. Histological analysis revealed marked improvement in the extent of fibrosis, a reduction in septal thickening, a decrease in fibroblast infiltration, and a reduction in alveolar inflammation compared with those in the untreated model group [33]. However, CGApotential role in modulating key profibrotic mediators in pulmonary fibrosis remains largely unexplored.

The present study investigated the potential of CGA to reverse or attenuate progressive tissue remodeling within a robust murine model of BLM-induced pulmonary fibrosis. Using histological, immunohistochemical, and gene expression analyses, we investigated its ability to attenuate inflammation, reduce collagen deposition, and modulate TGF-β expression, supporting its potential as a therapeutic candidate for FPI.

## 2. Materials and Methods

### 2.1. Ethical Considerations and Experimental Animals

All experimental procedures were carried out in strict compliance with international ethical standards for animal research and applicable national legislation. The study protocol was reviewed and approved by the Institutional Ethics Committee for the Care and Use of Animals (CIAUC) of the Faculty of Medicine and Surgery of the Autonomous University Benito Juárez of Oaxaca (registration number: 0047-CEI-2022). Sixteen male CD1 strain mice, aged 6–8 weeks and weighing 34–42 g, were used. The mice were acquired from the Laboratory Animal Production and Experimentation Unit of the Center for Research and Advanced Studies of the National Polytechnic Institute (UPEAL-CINVESTAV-IPN). Prior to the start of the experimental interventions, the rodents underwent a two-week acclimatization period. The animals were housed under controlled animal facility conditions, with a constant temperature of 22 ± 3 °C, an automated 12 h light/dark cycle, and access to water and food ad libitum.

### 2.2. Experimental Design and Idiopathic Pulmonary Fibrosis Model

For the development of IPF, a murine model based on the controlled and continuous administration of BLM via osmotic minipumps was used, adapting the methodology described previously [34,35,36]. The study population (*n* = 16 male mice) was randomly divided into four equidistant experimental conditions (*n* = 4 per group):

- Control (CT): Healthy subjects exposed only to sham surgical interventions.
- Chlorogenic acid (CGA): Animals that received only phenolic compounds.
- Bleomycin (BLM): Group in which fibrotic pulmonary pathology was induced.
- BLM + CGA: Subjects with active induction of IPF who underwent subsequent treatment with the compound.

**Figure 1:**
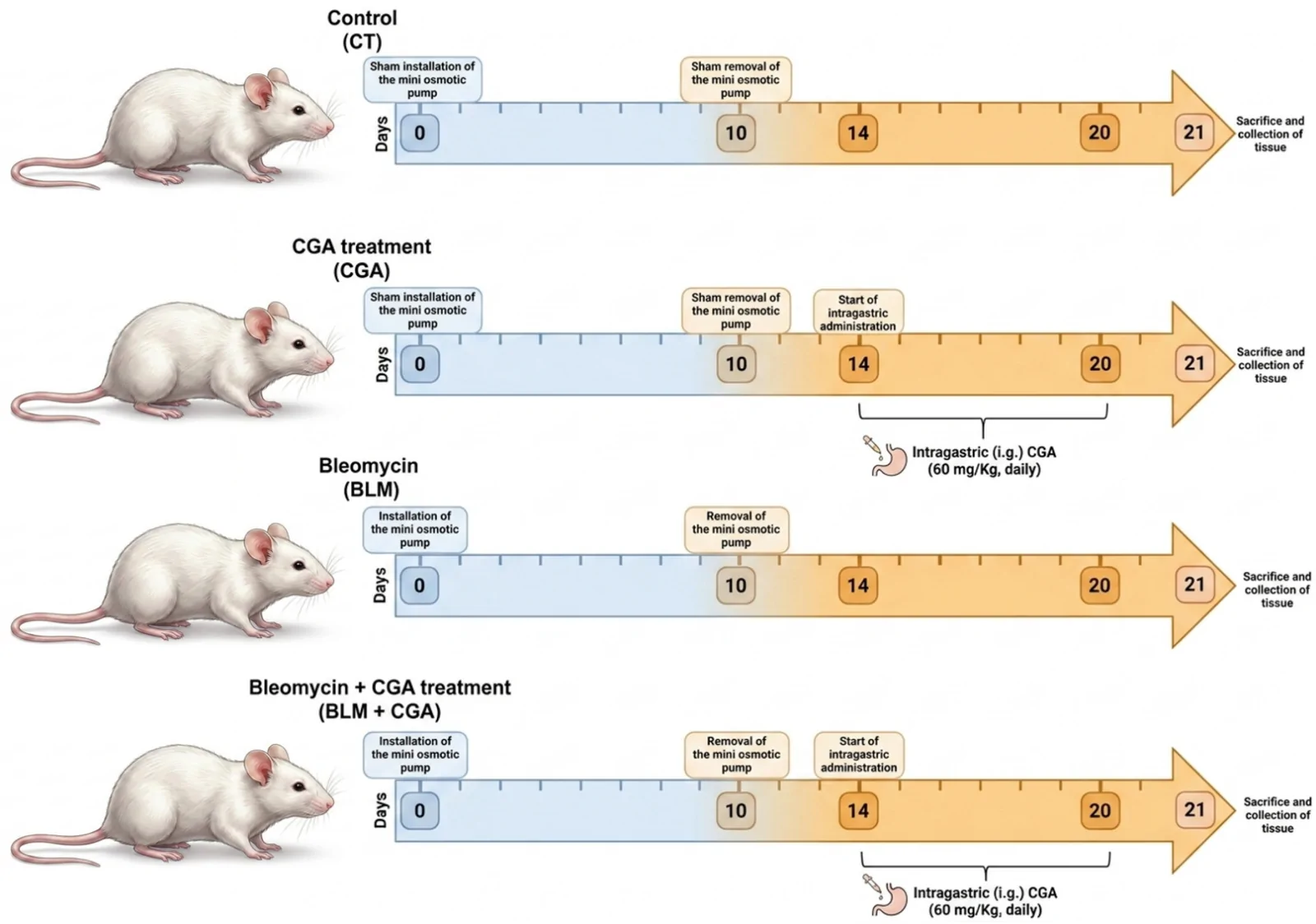
Schematic representation of the experimental design. Murine model of bleomycin (BLM)-induced idiopathic pulmonary fibrosis and chlorogenic acid (CGA) administration. On day 0, an osmotic minipump loaded with BLM (or saline for sham groups) was subcutaneously implanted to initiate a continuous 10-day systemic infusion. On day 10, the osmotic minipumps were surgically removed. On day 14, daily intragastric administration of CGA (60 mg/kg) or vehicle was initiated and continued until day 20. On day 21, mice from all experimental groups were sacrificed, and lung tissue samples were collected for subsequent histopathological, morphometric, and molecular analyses. BLM, bleomycin; CT, control; CGA, chlorogenic acid.

The initial operative phase (day 0) consisted of the subcutaneous placement of osmotic minipumps (model 1007D, ALZET; DURECT, Cupertino, CA, USA) in the interscapular region of the BLM and BLM + CGA groups under isoflurane-induced inhalational anesthesia. These devices delivered BLM (BLEOCEL, Celon Labs, Hyderabad, India) at a constant infusion rate of 0.5 μL/h until a final concentration of 100 U/kg was reached after one week. The baseline groups (CT and CGA) underwent a sham procedure under the same anesthetic parameters. Following the manufacturer’s removal guidelines, the micro-osmotic instrument was surgically removed on day 10 of the protocol in the stimulated groups, while sham extraction surgeries were performed concurrently in the control groups. The therapeutic window with CGA (C3873; Sigma Aldrich, St. Louis, MO, USA) began on day 14 with daily intragastric administration at a dosage of 60 mg/kg (vehicle: sterile water), which continued uninterrupted until day 20 in the corresponding groups (CGA and BLM + CGA). Finally, on day 21 of the experimental scheme, the animals were euthanized under anesthesia for dissection and immediate preservation of lung tissue for subsequent analysis. The complete temporal progression of these phases is illustrated graphically in Figure 1.

### 2.3. Obtaining, Processing, and Preservation of Lung Tissue Samples

To diversify the subsequent analyses, the lung tissues were anatomically divided into the following lobes:

The left lung lobe was designated for molecular assays; for this purpose, it underwent flash cryopreservation using a 2-methylbutane bath cooled to −80 °C and was kept deep frozen until use.

The right lung lobe was preserved for histological and immunohistochemical evaluations. This tissue was initially immersed in a formalin fixative solution (4% formaldehyde) for a minimum of 24 h. After fixation, the sample was subjected to an automated tissue dehydration protocol by immersion in increasing concentrations of ethyl alcohols (70%, 80%, 90%, and 96%), followed by intermediate clearing in an ethanol–xylene mixture (1:1 ratio) and final baths in pure xylene, with each immersion programmed for 60 min. The tissue was subsequently infiltrated and embedded in compact blocks of refined paraffin at a controlled temperature of 56 °C. Serial cross-sections were prepared using a rotary microtome (model RM2125RTS, Leica; Wetzlar, Germany). For cell morphology and general histology analyses, 5-μm-thick sections were obtained and mounted on gelatin-coated slides. In the case of immunohistochemical determinations, thinner sections of 3 μm were generated and strategically collected on silanized slides to optimize tissue adherence.

### 2.4. Histopathological Analysis

#### 2.4.1. Hematoxylin and Eosin (H&E) Staining

Histological abnormalities in the lung parenchyma were evaluated using routine hematoxylin and eosin (H&E) staining. After thermal deparaffinization at 56 °C, the tissue sections were gradually rehydrated by serial immersion in xylene, a xylene–ethanol mixture, and descending ethanol gradients (from 96% to 70%), remaining in each solution for 30 s before a final wash with running water. Nuclei were stained by incubating the preparations in Harris hematoxylin (738, HYCEL, Zapopan, Jalisco, Mexico) for 10 min. After a brief immersion in acid–alcohol and a fixation process in ammonia solution, a counterstain with yellowish eosin (688, HYCEL, Zapopan, Jalisco, Mexico) was performed for 10 min. Finally, the samples were dehydrated by immersion in increasing concentrations of ethanol and xylene. The cleared samples were mounted in synthetic resin and subsequently examined using a CARL ZEISS Primo Star optical microscope (CARL ZEISS, Jena, Germany) at 20× magnification (scale bar: 100 μm).

#### 2.4.2. Masson’s Trichrome Staining

ECM remodeling and collagen deposition were assessed using a commercial Masson’s trichrome kit (HT15, Sigma-Aldrich, St. Louis, MO, USA). After being oven deparaffinized at 56 °C, the tissue slides were rehydrated by immersion in xylene, xylene–ethanol, and decreasing amounts of ethanol (96% to 70%) for 30 s before being washed with tap water. The sample sections were then postfixed in Bouin’s solution at 56 °C for 1 h, followed by sequential staining with Weigert’s iron hematoxylin (20 min) and Biebrich’s acid fuchsin-scarlet (10 min). After differentiation in phosphotungstic/phosphomolybdic acid for 20 min, the fibers were counterstained with aniline blue for 1.5 h. The slides were then stabilized in 1% acetic acid for 15 s, dehydrated, cleared, and covered with coverslips. Photomicrographs captured at 20× magnification (Primo Star, CARL ZEISS; scale bar: 100 μm). Simultaneously, a modified Ashcroft index (ranging from 0 (normal architecture) to 8 (complete fibrous obliteration) was used for semiquantitative classification of the degree of fibrosis [37,38]. Scoring was performed blindly by two independent researchers, who averaged 20 random microscopic fields to obtain a representative value for each mouse.

#### 2.4.3. Digital Image Morphometry and Quantification Parameters

High-resolution photomicrographs (20× magnification) were analyzed using ImageJ v.2.3.0/1.53t software (US National Institutes of Health, Bethesda, MD, USA). For each biological subject (*n* = 4 per group), 20 randomly selected microscopic fields were evaluated per lung section.

To quantify the remaining alveolar area (%) and total cell count, H&E-stained images were converted into an RGB stack, and a fixed threshold was applied to isolate the tissue parenchyma from empty alveolar spaces. Cell numbers were automatically quantified using the “Analyze Particles” plugin after establishing a standardized threshold. For collagen-positive area (%) in Masson’s trichrome staining and protein expression positivity in immunohistochemistry (TGF-β1 and α-SMA), the “Color Deconvolution” plugin or thresholds in the HSB color space were used to isolate specific color signals (blue for collagen, brown for DAB). Fixed, standardized threshold limits were established for all images of each staining type to avoid subjective manual adjustments. Batch correction methods were not required, as all histological and immunohistochemical procedures were performed simultaneously in uniform batches, and image acquisition was carried out with constant light exposure and camera settings.

#### 2.4.4. Histopathological Evaluation via the Modified Ashcroft Score

To semi-quantitatively assess the severity and progression of IPF, the structural integrity of the lung parenchyma—visualized using Masson’s trichrome staining—was classified according to the modified Ashcroft scoring system. Histological changes in lung architecture were graded on a numerical scale from 0 to 8 based on the following criteria: a score of 0 indicates completely normal lung morphology; a score of 1 indicates minimal fibrous thickening of alveolar or bronchiolar walls; scores of 2 to 3 denote moderate wall thickening without evident damage to lung architecture; scores of 4 to 5 represent increased fibrosis with definite damage to lung structure and the formation of fibrous bands or small fibrous masses; scores of 6 to 7 indicate severe structural distortion and large fibrous areas; and a score of 8 denotes total fibrous obliteration of the microscopic field.

To eliminate potential observer bias, this histopathological scoring was performed in a strictly blinded manner by two independent researchers who were completely unaware of the experimental design. For each biological subject (*n* = 4 per group), the assessment was conducted on 20 randomly selected, non-overlapping microscopic fields captured at 20× magnification; subsequently, the individual field scores were averaged to obtain a single, highly representative numerical value for each mouse.

### 2.5. Immunohistochemistry

Three-micron-thick tissue sections were deparaffinized, rehydrated, and incubated in citrate buffer (pH 6.0) using a pressure cooker for antigen retrieval. The sections were then permeabilized with phosphate-buffered saline containing 0.1% Triton X-100 (PBS-T) for 10 min. Endogenous peroxidase activity was blocked with 6% hydrogen peroxide in methanol for 30 min at room temperature, after which the nonspecific sites were blocked with 3% bovine serum albumin (BSA) in 1× PBS for 1 h at room temperature. The tissues were subsequently incubated for 16 h at 4 °C with the following primary antibodies diluted in 1% BSA: rabbit polyclonal anti-α-SMA IgG (1:800; Cat. No. 55135-1-AP; Proteintech, Chicago, IL, USA), and rabbit polyclonal anti-TGF-β1 IgG (1:300; Cat. No. ab92486; Abcam, Cambridge, UK). After being washed with PBS, the sections were incubated with a goat polyclonal anti-rabbit IgG conjugated to horseradish peroxidase (HRP) secondary antibody (1:600; Cat. No. ab6721; Abcam, Cambridge, UK) for 1 h at 37 °C. To validate staining specificity and rule out non-specific background signals, negative controls were performed by omitting the functional primary antibodies and substituting them with a normal rabbit IgG polyclonal isotype control (Cat. No. ab37415; Abcam, Cambridge, UK) at concentrations and experimental settings identical to those of the primary antibodies. The signal was detected using a diaminobenzidine substrate kit (DAB SK-4100; Vector Laboratories, Burlingame, CA, USA), counterstained with Harris hematoxylin, and examined under a CARL ZEISS Primo Star optical microscope at 20× magnification. Digitized photomicrographs were captured under uniform light exposure and white-balance settings, and the percentage of positively immunolabeled surface area was subsequently quantified using the standardized digital parameters described in Section 2.4.3.

### 2.6. RNA Extraction and qRT–PCR

Quantitative real-time PCR (RT–qPCR) was used to evaluate gene expression profiles in lung tissue. Total RNA isolation was performed using the Quick-RNA Miniprep Plus system (Cat. No. R1054; Zymo Research, Los Angeles, CA, USA). Complementary DNA (cDNA) synthesis was then carried out by reverse transcription of the purified RNA templates using a RevertAid First Strand kit (Cat. No. K1622; Thermo Scientific, Waltham, MA, USA). Amplicons were amplified and detected using SYBR Green PCR Master Mix (Cat. No. K0172; Thermo Scientific) on a StepOne Real-Time PCR instrument (Applied Biosystems, Waltham, MA, USA; Model 4376600). The specific oligonucleotide sequences of primers used for target gene amplification are listed in Table 1. The thermocycling profile consisted of initial denaturation followed by 40 cycles at 95 °C for 15 s, 60 °C for 30 s, and 72 °C for 30 s. The relative abundance of the target mRNA was determined using the comparative method 2−ΔΔCT, employing β-actin as the endogenous reference gene for normalization.

### 2.7. Statistical Analysis

Data derived from histological and immunohistochemical analyses were initially quantified using ImageJ v.2.3.0/1.53t software. These results, along with the relative expression values determined by qRT–PCR, are presented as the mean ± SD. To determine the statistical significance between groups, GraphPad Prism 8.0 software (GraphPad, San Diego, CA, USA) was used. One-way analysis of variance (ANOVA) supplemented with Tukey’s post hoc test was performed. The threshold for establishing significant differences was set at *p* < 0.05.

## 3. Results

### 3.1. CGA Attenuates Histological Alterations Associated with BLM-Induced Pulmonary Fibrosis in Mice

**Figure 2:**
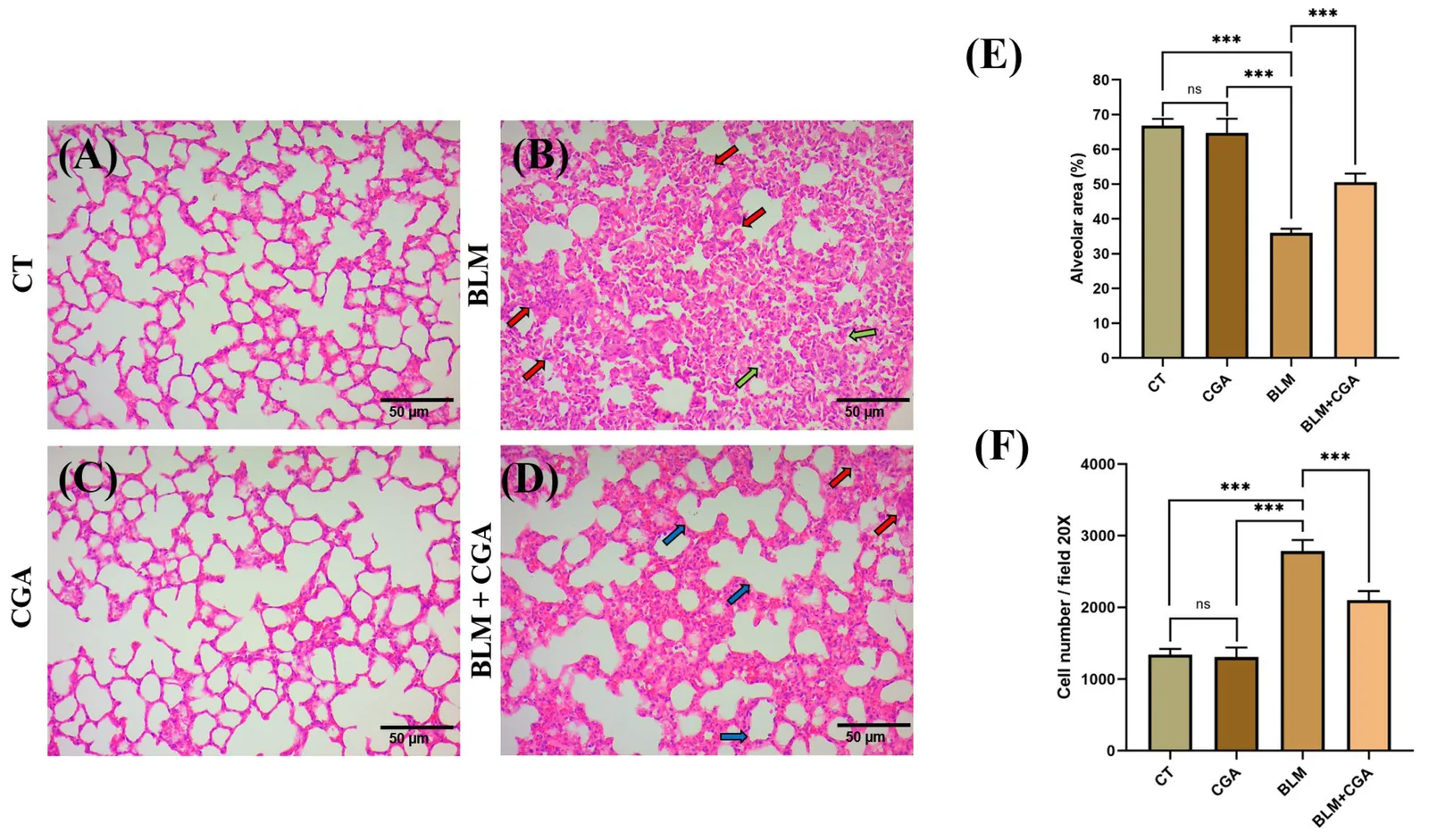
Histopathological and morphometric evaluation of lung tissue in the different study groups. (**A**–**D**) Representative photomicrographs of lung tissue stained with hematoxylin and eosin (H&E) corresponding to the groups: (**A**) CT, (**B**) BLM, (**C**) CGA, and (**D**) BLM + CGA. In the BLM group (**B**), green arrows indicate areas of marked thickening of the alveolar septa, and red arrows show dense cell infiltration. In the BLM + CGA group (**D**), red arrows indicate remaining cell infiltration, while blue arrows demonstrate prominent restoration of alveolar spaces. Scale bar = 50 μm; magnification = 20×. (**E**) Morphometric analysis of the percentage of alveolar area. (**F**) Quantitative analysis of cell number per field. The data are presented as the mean ± SD (*n* = 4). Statistical analysis was performed using one-way ANOVA followed by Tukey’s multiple comparison test. Image analysis was performed using ImageJ 1.53t software. ns: not significant; *** *p* < 0.001. CT: control; CGA: chlorogenic acid; BLM: bleomycin.

To evaluate the protective effect of CGA against BLM-induced progressive pulmonary fibrosis, histopathological analysis of lung tissue was performed using H&E staining (Figure 2). As shown in Figure 2A,C, histopathological examination of the control (CT) group and the group treated solely with CGA revealed a well-defined lung architecture. These groups exhibited normal lung parenchyma, characterized by thin alveolar septa, clear, well-defined alveolar spaces, and the absence of abnormal cellular accumulations or structural damage.

In contrast, BLM administration (Figure 2B) induced severe and extensive parenchymal distortion. This group exhibited classic features of progressive fibrotic lung injury, including marked thickening of the alveolar septa, leading to a drastic loss of functional airspaces, as indicated by the green arrows. Furthermore, dense cellular infiltration characterized by a prominent accumulation of nuclei was observed, as indicated by the red arrows. Morphometric quantification confirmed these structural alterations, showing a significant reduction in the percentage of alveolar area (Figure 2E, *p* < 0.001) and a sharp increase in the total cell count per field (Figure 2F, *p* < 0.001) compared to the CT and CGA groups.

It is worth noting that CGA treatment significantly mitigated the histopathological deterioration induced by BLM. The BLM + CGA group (Figure 2D) showed substantial preservation of lung structure, exhibiting evident restoration of alveolar spaces, as indicated by the blue arrows. Although mild residual cellular infiltration persisted in slightly thickened septa in certain localized areas (indicated by red arrows in Panel D) compared to healthy controls (CT and CGA), the overall tissue architecture improved significantly relative to the BLM group. This histological recovery was strongly supported by morphometric data, which demonstrated a significant increase in the remaining alveolar area (Figure 2E, *p* < 0.001) and a marked decrease in total cellular infiltration per field (Figure 2F, *p* < 0.001) compared with the BLM group. Taken together, these results suggest that CGA administration exerts protective and therapeutic effects against BLM-induced lung damage.

### 3.2. CGA Reduces Collagen Deposition in BLM-Induced Pulmonary Fibrosis in Mice

**Figure 3:**
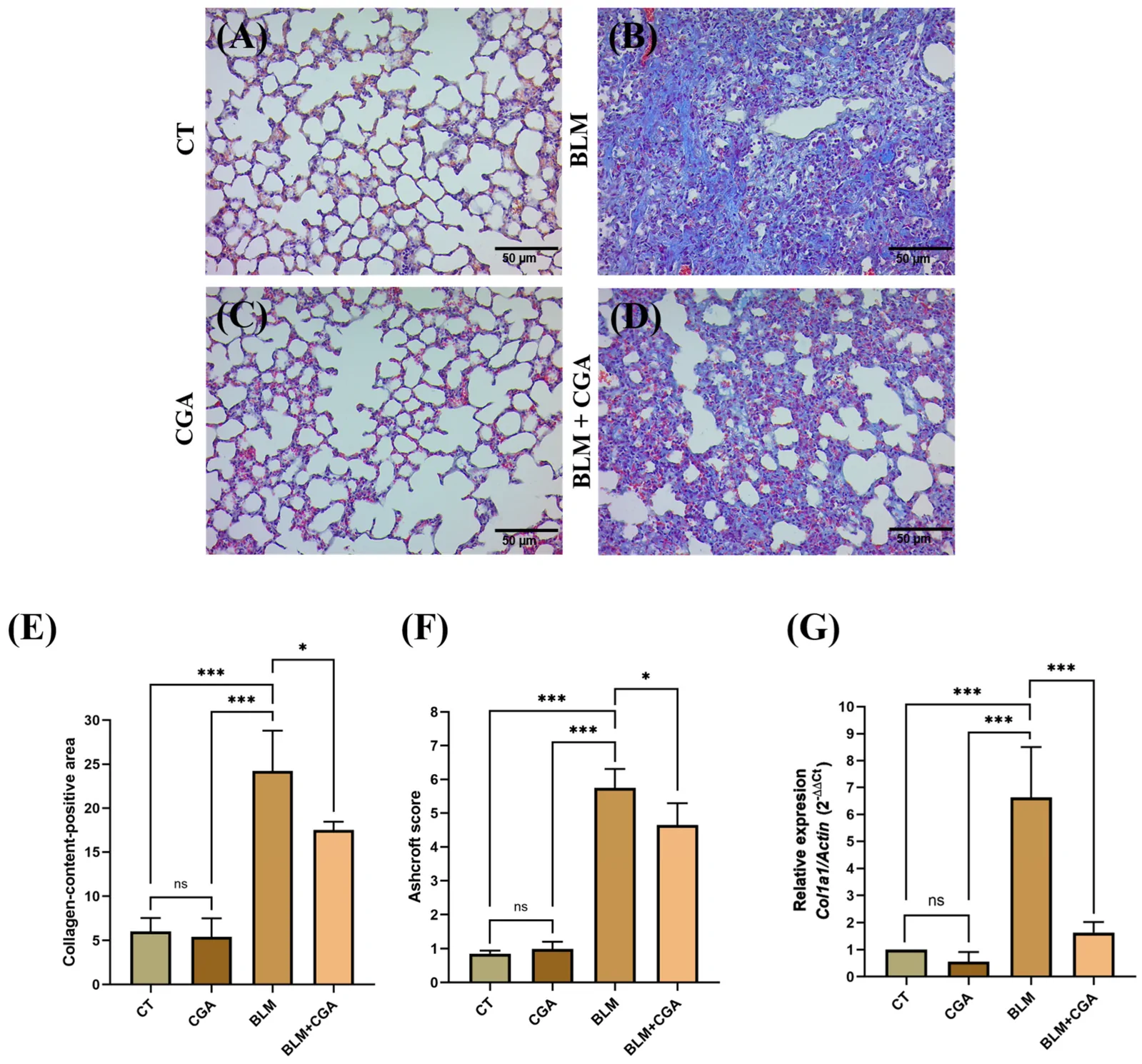
Evaluation of lung fibrosis and collagen deposition in the different study groups. (**A**–**D**) Representative photomicrographs of lung tissue stained with Masson’s trichrome corresponding to the following groups: (**A**) CT, (**B**) BLM, (**C**) CGA, and (**D**) BLM + CGA. Scale bar = 50 μm; magnification = 20×. Collagen deposition is shown in blue. (**E**) Percentage of collagen-positive area in lung tissue for each group. (**F**) Histopathological assessment of lung fibrosis using the Ashcroft score. (**G**) Relative Col1a1 mRNA expression levels determined by real-time quantitative PCR. The data are presented as the mean ± SD (*n* = 4). Statistical analysis was performed using one-way ANOVA followed by Tukey’s multiple comparison test. Image analysis was performed using ImageJ 1.53t software. ns: not significant; * *p* < 0.05; *** *p* < 0.001. CT: control; CGA: chlorogenic acid; BLM: bleomycin.

Masson’s trichrome staining, which stains collagen fibers an intense blue, was used to evaluate ECM remodeling and determine the spatial distribution of fibrotic lesions induced by continuous BLM administration (Figure 3). As shown in Figure 3A,C, the healthy control groups (CT and CGA) exhibited basal collagen expression, with a fully preserved lung parenchyma.

In contrast, the BLM-treated group showed a pronounced accumulation of dense collagen matrices (Figure 3C) compared to the CT and CGA control groups. This abnormal deposition was predominantly distributed within the diffuse alveolar interstitium and subpleural regions, resulting in extensive thickening of the alveolar walls and massive parenchymal consolidation. On the other hand, therapeutic intervention with CGA notably attenuated both the staining intensity and the structural extent of these collagen fiber deposits in the BLM + CGA group (Figure 3D).

Image analysis for the morphometric quantification of the collagen-positive area corroborated these qualitative findings, revealing a statistically significant increase in the percentage of collagen content in the BLM group compared to the CT and CGA groups; this increase was effectively suppressed by the co-administration of CGA, as observed in the BLM + CGA group (Figure 3E). Image analysis performed to determine the fibrosis score according to Ashcroft criteria revealed a significant increase in the BLM group compared to the CT and CGA control groups. Compared to the BLM group, the BLM + CGA group showed a significant reduction in the Ashcroft score (Figure 3F). To further investigate the effect of CGA on collagen deposition, Col1a1 mRNA expression levels in lung tissue were evaluated using qRT-PCR. The results indicated that compared with the CT and CGA groups, BLM treatment significantly increased Col1a1 expression. Conversely, CGA treatment was associated with a significant reduction in Col1a1 expression in the BLM + CGA group (Figure 3G). Taken together, these findings indicate that CGA attenuates BLM-induced pulmonary fibrosis by reducing collagen deposition and the expression of key ECM components in lung tissue.

### 3.3. CGA Reduces the Expression of TGF-β1, a Key Mediator of BLM-Induced Pulmonary Fibrosis in Mice

**Figure 4:**
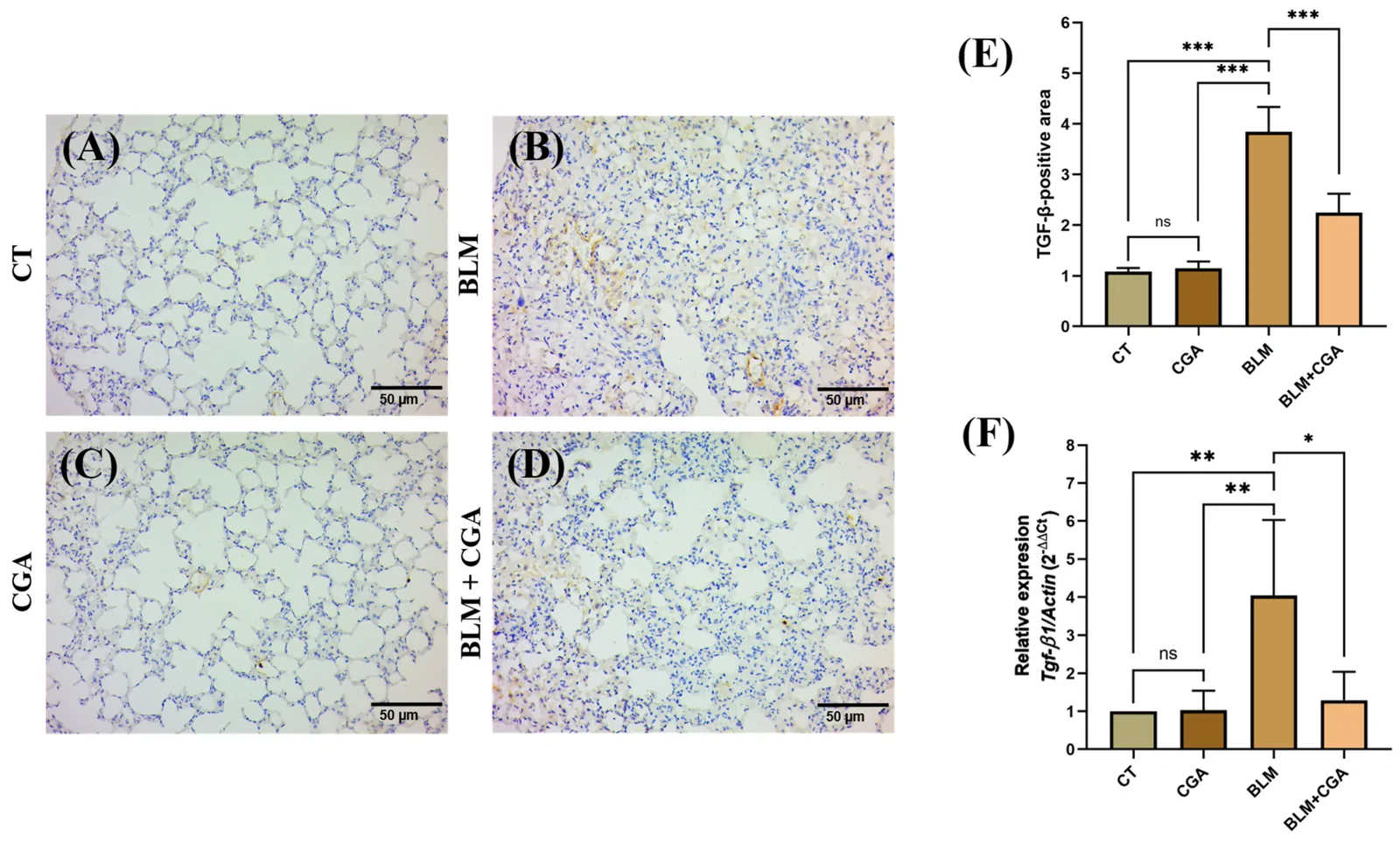
TGF-β1 expression in lung tissue in the different study groups. (**A**–**D**) Representative immunohistochemical images of TGF-β1 expression in lung tissue from the following groups: (**A**) CT, (**B**) BLM, (**C**) CGA, and (**D**) BLM + CGA. Scale bar = 50 μm; magnification = 20×. Positive staining is shown in brown. (**E**) Percentage of the area in which lung tissue was positive for TGF-β1 expression in each group. (**F**) Relative TGF-β1 mRNA expression levels determined by real-time quantitative PCR. The data are presented as the mean ± SD (*n* = 4). Statistical analysis was performed using one-way ANOVA followed by Tukey’s multiple comparison test. Image analysis was performed using ImageJ 1.53t software. ns: not significant; * *p* < 0.05; ** *p* < 0.01; *** *p* < 0.001. CT: control; CGA: chlorogenic acid; BLM: bleomycin.

TGF-β1, recognized as a pivotal factor in the triggering and perpetuation of fibrotic remodeling, acts as a potent mediator of myofibroblast activation and excessive ECM synthesis, key features of the fibrotic process. Because of its central influence on progression of the lung fibrotic process, TGF-β1 expression was analyzed by immunohistochemistry to better understand its involvement in the observed histological changes and to determine the efficacy of experimental treatment with CGA on this profibrotic molecule. The control groups (CT and CGA) had low levels of TGF-β1 expression, indicating the basal expression of this marker (Figure 4A,C). In contrast, the group exposed to BLM exhibited a robust upregulation of TGF-β1 immunoreactivity, predominantly in fibrotic areas (Figure 3B). On the other hand, compared with the mice exposed to BLM alone, the therapeutic intervention combining BLM and CGA (BLM + CGA) exhibited a striking suppression of the TGF-β1-positive cellular extension (Figure 4D). Quantitative analysis of immunohistochemical images supported these observations, revealing a significant increase in the area of TGF-β1-positive cells in the BLM-treated group compared with that in the control groups (CT and CGA). In contrast, compared with BLM treatment, treatment with CGA resulted in a significant decrease in these positive areas (Figure 4E). To further elucidate the regulatory influence of CGA on the profibrotic cascade, the transcriptional modulation of TGF-β1 was evaluated in the lung parenchyma via RT-qPCR. Our quantitative analysis demonstrated that, relative to the healthy control cohort, BLM administration triggered a robust upregulation of relative TGF-β1 mRNA transcript levels. However, a significant decrease in TGF-β1 mRNA expression was observed in the BLM + CGA group compared with that in the BLM-only group (Figure 4F). These findings suggest that CGA exerts an antifibrotic effect that is associated with reduced TGF-β1 expression, which may contribute to decreased myofibroblast activation and decreased fibrosis progression. These findings support the potential of CGA as a therapeutic strategy to mitigate the pro-fibrotic effects of TGF-β1 in lung fibrosis models.

### 3.4. CGA Reduces the Expression of the Myofibroblast Marker α-SMA in BLM-Induced Pulmonary Fibrosis in Mice

**Figure 5:**
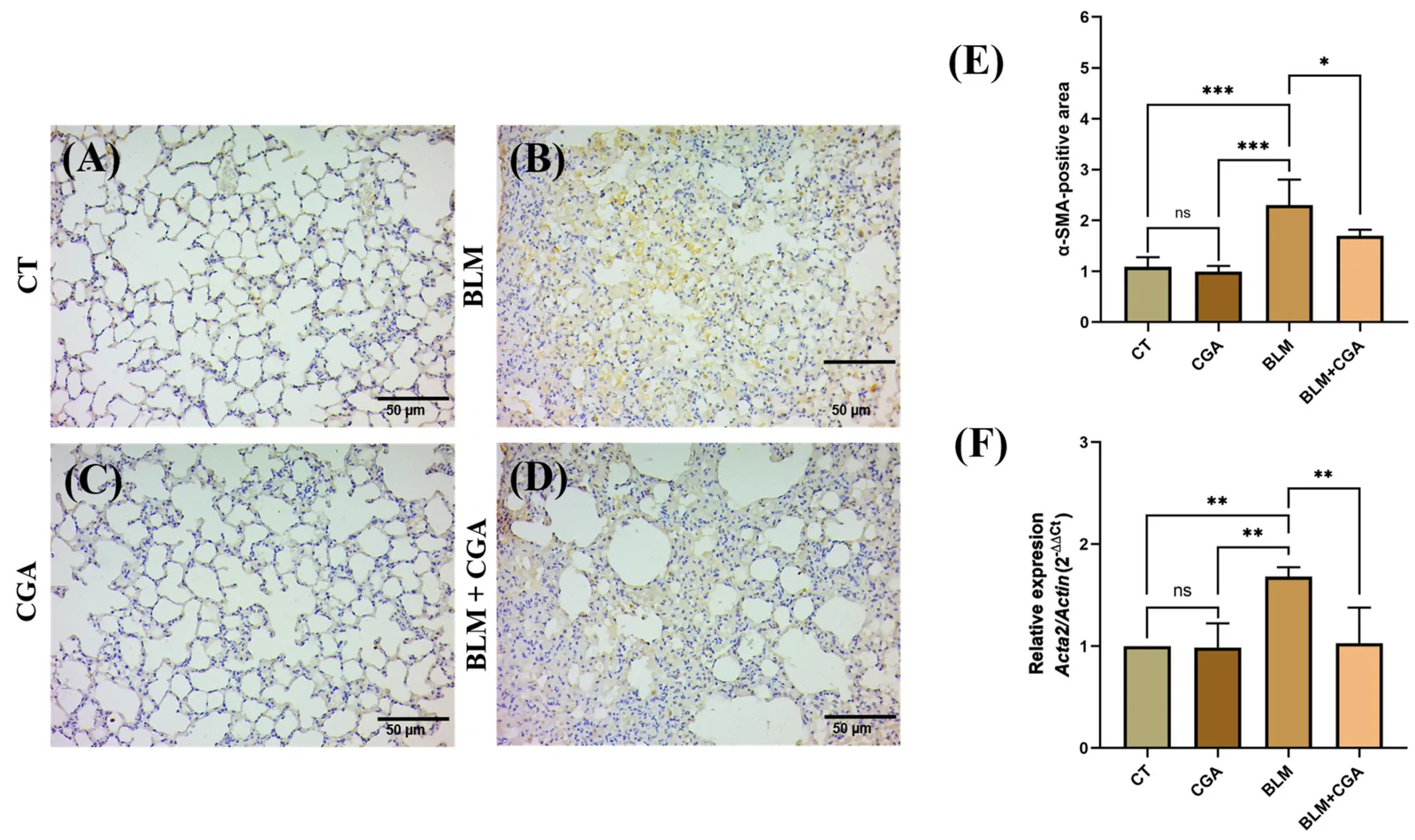
α-SMA expression in lung tissue in the different study groups. (**A**–**D**) Representative immunohistochemical images of α-SMA in lung tissue from the following groups: (**A**) CT, (**B**) BLM, (**C**) CGA, and (**D**) BLM + CGA. Scale bar = 50 μm; magnification = 20×. Positive staining is shown in brown. (**E**) Percentage of the α-SMA-positive area in the lung tissue for each group. (**F**) Relative α-SMA (*Acta2*) mRNA expression levels determined by real-time quantitative PCR. The data are presented as the mean ± SD (*n* = 4). Statistical analysis was performed using one-way ANOVA followed by Tukey’s multiple comparison test. Image analysis was performed using ImageJ 1.53t software. ns: not significant; * *p* < 0.05; ** *p* < 0.01; *** *p* < 0.001. CT: control; CGA: chlorogenic acid; BLM: bleomycin.

Given that myofibroblasts serve as pivotal drivers and are orchestrating the accumulation of ECM in fibrotic processes, an immunohistochemical analysis was performed to determine the levels of α-SMA, which serves as a characteristic classic indicator of their activation state. The control groups (CT and CGA) showed low levels of α-SMA positivity, reflecting the basal expression of this marker (Figure 5A,C). On the other hand, the BLM-treated group exhibited a marked increase in α-SMA positivity, particularly in fibrotic areas (Figure 5B). However, compared with the BLM-only group, mice treated with CGA (BLM + CGA) presented an evident reduction in α-SMA-positive areas (Figure 5D). Quantitative analysis of immunohistochemical images confirmed these observations, revealing a significant increase in α-SMA-positive areas in the BLM group compared with those in the CT and CGA control groups. In contrast, compared with BLM treatment, treatment with CGA (BLM + CGA) resulted in a significant decrease in these areas (Figure 5E). To delve deeper into the impact of CGA on α-SMA transcript levels, the relative mRNA abundance of this marker in lung tissue was measured utilizing RT-qPCR analysis. Compared with the CT and CGA control treatments, the BLM treatment resulted in a substantial elevation in α-SMA mRNA. In contrast, compared with the BLM group, the BLM + CGA group had significantly lower α-SMA mRNA expression (Figure 5F). These results suggest that CGA treatment modulates BLM-induced myofibroblast activation, as evidenced by the reduction in α-SMA-positive areas and their mRNA levels. These findings highlight the ability of CGA to limit the activation of these key cells in the development of pulmonary fibrosis.

## 4. Discussion

This research aimed primarily to investigate the therapeutic efficacy of CGA against progressive tissue remodeling in a murine model of established pulmonary fibrosis. Our findings demonstrate that treatment with 60 mg/kg CGA significantly reversed BLM-induced lung damage by limiting alveolar architectural collapse, cellular infiltration, and aberrant ECM accumulation.

A key methodological advantage of the present study lies in the strategic timing of the intervention within the therapeutic window. In the subcutaneous osmotic minipump model, the pathological cascade comprises distinct sequential phases: an initial acute inflammatory phase peaking within the first 7 days, followed by an active fibroproliferative phase between days 7 and 14, culminating in mature ECM deposition and parenchymal remodeling by day 21 and 28 [36,39,40]. Unlike experimental designs that initiate the administration of natural compounds simultaneously with or immediately after BLM injury—thereby primarily assessing anti-inflammatory or cytoprotective preventive effects—our therapeutic protocol using CGA was deliberately delayed until day 14. By this time, the acute inflammatory response has largely subsided, and the fibrotic microenvironment, characterized by myofibroblast activation and collagen deposition—is already firmly established [36,39,41]. Consequently, the significant architectural restoration and molecular downregulation observed on day 21 suggest that CGA exerts an active antifibrotic effect that can interrupt and modulate an ongoing fibrotic process, rather than merely mitigating the initial inflammatory triggers.

Our histopathological evaluations confirmed that BLM triggers severe parenchymal remodeling. This model characteristically exhibits alveolar septal thickening and dense cell infiltration [36,39]. In contrast, post-CGA treatment successfully reversed these architectural abnormalities. The remarkable recovery of the alveolar area percentage and the reduction in total cell density demonstrate its protective effect. These improvements correlate with previous reports demonstrating the anti-inflammatory properties of dietary polyphenols such as CGA in experimental models of pulmonary fibrosis [33,42]. Prior evidence has shown that CGA administration significantly attenuates progressive lung damage in a pulmonary fibrosis model established via intratracheal instillation of BLM in BALB/c mice. Histologically, treatment with different concentrations of CGA markedly mitigated the typical fibrotic alterations caused by BLM, such as alveolar septal thickening, fibroblast proliferation, alveolar inflammation, and lymphoid invasion. These pathological changes were reduced in a dose-dependent manner, with the highest dose (60 mg/kg) being most effective at restoring near-normal lung morphology [33]. Another similar study revealed that after 21 days of treatment, CGA significantly reduced inflammatory cell infiltration and pulmonary interstitial thickening in a model of pulmonary fibrosis elicited by intratracheal instillation of BLM in C57BL/6J mice [42].

ECM protein hyperproduction serves as a critical pathophysiological signature of progressive lung fibrogenesis [43,44]. In our study, Masson’s trichrome staining revealed that CGA significantly suppressed excessive collagen deposition, although progressive IPF involves a complex network of multi-component ECM proteins—including fibronectin, tenascin-C, periostin, proteoglycans, matrix metalloproteinases, and fibrillar collagens [44,45,46,47]. The type I collagen alpha-1 chain (encoded by the *Col1a1* gene) is one of the primary and most abundant structural components, responsible for irreversible parenchymal distortion and restrictive mechanical lung failure [44,48,49]. In our model, the marked decrease in *Col1a1* mRNA transcripts induced by CGA treatment was associated with a visible reduction in the macrostructural density of collagen fibers—as evidenced by Masson’s trichrome staining—and a concomitant decrease in the Ashcroft structural score.

Previous research has suggested that CGA is capable of reducing or halting the active synthesis of collagen fibers in fibrotic processes [32,42,50]. For example, in a CCl4-induced liver fibrosis model in male Sprague–Dawley rats, treatment with different concentrations of CGA (15, 30, and 60 mg/kg) markedly decreased the area of collagen fibers, as revealed by Masson’s trichrome staining. Interestingly, the highest concentration showed the most potent antifibrotic effect [32]. Similarly, the results of a study in a murine model of isoproterenol-induced cardiac hypertrophy demonstrated that CGA intervention significantly suppresses the pathological accumulation of collagen fibers in cardiac tissue. Histological evaluation using Picrosirius red staining revealed that this decrease in ECM deposition correlated directly with potent molecular inhibition of type I collagen (*COL1A1*), the main structural component of fibrotic scarring [51]. In terms of pulmonary fibrosis, histological analysis with Masson’s trichrome staining demonstrated that CGA treatment significantly decreases aberrant collagen deposition in the lung tissue of mice with BLM-induced fibrosis [42]. These findings suggest that CGA may attenuate the pulmonary fibrogenic process, confirming its potential as a therapeutic agent for pulmonary fibrosis through the improvement of histological parameters representative of the disease.

Our results align with the known ability of CGA to block fibrogenic cascades in other organs [29]. At the signaling level, TGF-β1 acts as the master orchestrator of fibrogenesis [14,15,16]. Our immunohistochemical and RT–PCR analyses demonstrated that CGA effectively reduced the protein and mRNA expression of TGF-β1. We hypothesize that this restriction of signaling led to a marked decrease in the expression of the myofibroblast marker α-SMA. By preventing the activation of α-SMA-positive cells, CGA acts directly on the central effector phase of pulmonary fibrosis.

Our findings align with earlier investigations demonstrating that CGA exerts a compelling anti-fibrotic action in hepatic tissue by fine-tuning the TGF-β1/Smad7 signaling cascade under the epigenetic control of miR-21. Specifically, CGA reduces the expression of TGF-β1 and its downstream signaling molecules, including the phosphorylated forms of Smad2 and Smad3, which are key in driving the phenotypic transition of hepatic stellate cells and the subsequent overproduction of ECM. Furthermore, CGA treatment increased the expression of Smad7, a negative regulator of the TGF-β1 cascade, indicating that CGA restored the balance of this signaling cascade, promoting the inhibition of fibrosis progression. On the other hand, CGA positively modulates MMP-9 levels and inhibits the expression of profibrotic mediators including α-SMA, CTGF, and TIMP-1, thereby facilitating ECM degradation [32]. Similarly, studies conducted in an IL-13-stimulated LX2 hepatic stellate cell model revealed that CGA markedly decreased the phosphorylation of Smad2/3 and the protein expression of TGF-β receptor I, key components involved in the activation of the fibrogenic pathway. Simultaneously, CGA promoted the expression of Smad7, an endogenous inhibitor of TGF-β-mediated signaling, thus contributing to the suppression of the activation of these pathways and the consequent reduction in the expression of profibrotic factors such as CTGF and α-SMA. These effects were corroborated in vivo in a murine model of Schistosoma japonicum infection, in which CGA treatment reduced Smad2/3 protein phosphorylation and TGF-β receptor I expression, which correlated with a significant decrease in hepatic fibrosis and inflammation [52].

Moreover, in a murine model of I/R kidney injury, CGA administration, especially at the highest dose (14 mg/kg), resulted in a significant, dose-dependent reduction in TGF-β1 mRNA abundance. This decrease in TGF-β1 expression was accompanied by a reduction in myofibroblast expansion (as measured by α-SMA) and the expression of mesenchymal markers such as vimentin, suggesting the inhibition of the EMT process and renal fibrosis [31]. Similarly, prolonged administration of CGA in a murine model of type 2 diabetes (db/db mice) significantly reduced TGF-β1 protein expression in the kidney, accompanied by a decrease in aldose reductase activity. These effects suggest that CGA may attenuate renal fibrosis processes associated with diabetic nephropathy by modulating key signaling pathways related to hyperglycemia-induced metabolic stress [53].

Furthermore, CGA has been shown to have protective effects against cardiac fibrosis. Available evidence has shown that CGA significantly reduces the content of α-SMA in the cardiac tissue of diabetic mice and in fibroblasts cultured under hyperglycemic conditions. In addition, CGA efficiently prevents p-Smad2/3 from translocating into the nucleus, thereby halting a pivotal step in the TGF-β pathway that drives fibrosis [54].

These multiorgan protective effects of CGA align precisely with recent and specific evidence generated from pulmonary pathophysiology models. In a comprehensive study using a murine model of BLM-induced IPF, oral administration of CGA effectively reversed parenchymal collapse, significantly reducing cellular infiltration and Ashcroft scores. At the transcriptional level, the therapeutic efficacy of CGA depends on the significant downregulation of key core genes, including the receptor tyrosine kinase Egfr, the matrix remodeling enzyme Mmp9, the core survival kinase Akt1, and the proinflammatory cytokine Il1b [50]. This simultaneous restriction of inflammatory and fibrogenic drivers directly complements the chronic progressive context explored in a model of diabetes-induced pulmonary fibrosis, which revealed that systemic administration of CGA upregulates the expression of Smad7, an endogenous negative regulator of the canonical TGF-β1 cascade, resulting in a marked downregulation of TGF-β1 and α-Sma at both the mRNA and protein levels in lung tissue. Interestingly, their work highlights that this lung-protective effect is highly potent even at lower doses (12.5 mg/kg) and operates through pathways that mitigate tissue remodeling independently of the compound’s systemic hypoglycemic properties [55]. In addition to the classical TGF-β1/Smad signaling cascade, the protection exerted by CGA in the lung has been closely linked to the resolution of intracellular stress and cell death networks. Through both in vivo and in vitro models, CGA was shown to act as a potent inhibitor of endoplasmic reticulum (ER) stress, an upstream driver that commonly orchestrates epithelial–mesenchymal transition (EMT) and chronic fibrotic remodeling. By specifically suppressing UPR sensor activation through the downregulation of phosphorylated PERK (p-PERK) and cleaved ATF-6, CGA treatment caused a dose-dependent reduction in the expression of the ER proapoptotic chaperones GRP78 and CHOP. Consequently, this restriction of sustained ER stress effectively halts the downstream cytotoxic apoptotic cascade, with a notable downregulation of cleaved caspase-12, -9, and -3 serving as evidence, along with the concomitant rescue of uncleaved PARP expression [33]. By demonstrating that CGA actively intercepts the Egfr/Mmp9/Akt1 axis during acute lung injury, restores the Tgf-b1/Smad7/a-Sma homeostatic balance in metabolic injury, and robustly targets the ER stress-mediated caspase cascade, these independent pulmonary reports strongly corroborate our observations, establishing a strong biological consensus that CGA functions as a highly reproducible, multitarget therapeutic candidate against progressive pulmonary fibrogenesis [33,50,55].

Despite the promising results obtained in this study, certain limitations must be acknowledged. First, although we confirmed that CGA effectively halts advanced stages of fibrosis—for instance, by reducing TGF-β1 and α-SMA levels—the exact initial point at which CGA binds to cells to trigger this protective response remains unknown. Second, while continuous BLM administration via osmotic minipumps provides a highly controlled, reliable, and reproducible model of severe parenchymal remodeling, our experimental design focused on an early treatment window (days 14 to 20); although evidence from this experimental model indicates that fibrosis is already established during this period, future research should evaluate CGA’s therapeutic efficacy over a longer chronic timeframe—such as at days 28 and 35—to determine its ability to reverse mature, fully established fibrotic matrices, which would more closely resemble human clinical scenarios. Furthermore, although the sample size per group was statistically sufficient to detect robust and highly significant changes across all histological and molecular parameters, expanding experimental cohorts in future protocols would further strengthen the data obtained in our study. Finally, we acknowledge that the absence of physiological assessments of lung function is a significant limitation of the present study. While such functional assessments are an important complement for fully characterizing the restrictive nature of IPF, it is important to highlight that our morphometric parameters such as the quantitative restoration of alveolar area serve as a robust structural indicator of tissue recovery. Nevertheless, future research could incorporate the study of lung mechanics to comprehensively validate the potential for physiological translation of CGA in the context of IPF. Likewise, although TGF-β1 expression was evaluated, the canonical and non-canonical signaling pathways located downstream in the signaling cascade were not investigated.

## 5. Conclusions

In conclusion, our study demonstrated that intragastric administration of 60 mg/kg CGA has a potent therapeutic effect on BLM-induced pulmonary fibrosis in mice (p. 1). By successfully preventing alveolar architectural collapse, reducing cell infiltration density, and suppressing aberrant collagen matrix deposition, CGA effectively preserved lung tissue integrity. Mechanistically, these structural improvements are driven by robust downregulation of the expression of the central pro-fibrotic mediator TGF-β1, downregulation of *Col1a1* transcription, and inhibition of α-SMA-positive myofibroblast activation. Consequently, these findings support the use of CGA as a highly promising, multitargeted natural compound and support its further evaluation as a safe dietary or pharmacological agent to attenuate the progression of interstitial lung diseases.

## Figures and Tables

**Table 1:** Primers used in qRT–PCR.

Gene	Direction	Primer Sequence (5′→ 3′)
*β-actin*	FW RV	CAGCCTTCCTTCTTGGGTATG GGCATAGAGGTCTTTACGGATG
*Tgf-β1*	FW RV	CCTGAGTGGCTGTCTTTTGA CGTGGAGTTTGTTATCTTTGCTG
*Acta2*	FW RV	TCAGGGAGTAATGGTTGGAATG GGTGATGATGCCGTGTTCTA

## Data Availability

The original data generated and presented in the study are included in the article. Further queries can be sent to the corresponding authors.

## Abbreviations

The following abbreviations are used in this manuscript:

α-SMA	Alpha-smooth muscle actin
Akt1	AKT serine/threonine kinase 1
ATF-6	Activating transcription factor 6
BLM	Bleomycin
cDNA	Complementary DNA
CGA	Chlorogenic acid
CHO	C/EBP homologous protein
COL1A1	Collagen type I alpha 1 chain
CT	Control
CTGF	Connective tissue growth factor
ECM	Extracellular matrix
EGFR	Epidermal growth factor receptor
EMT	Epithelial–mesenchymal transition
ER	Endoplasmic reticulum
ERK	Extracellular signal-regulated kinases
GRP78	Glucose-regulated protein 78
H&E	Hematoxylin and eosin
I/R	Ischemia/reperfusion
Il1b	Interleukin 1 beta
ILDs	Interstitial lung diseases
IPF	Idiopathic pulmonary fibrosis
MAPK	Mitogen-activated protein kinase
MCP-1	Monocyte chemoattractant protein-1
MMP9	Matrix metallopeptidase 9
NF-κB	Nuclear factor kappa B
PARP	Poly (ADP-ribose) polymerase
PI3K/Akt	Fosfoinositol 3-quinasa/Proteína quinasa B
p-PERK	Phosphorylated PKR-like ER kinase
qRT–PCR	Quantitative real-time PCR
TGF-β1	Transforming growth factor-beta 1
TIMP-1	issue inhibitor of metalloproteinases 1
TLR-4	Toll-like receptor 4
TNF-α	Tumor necrosis factor alpha
